# Factors Affecting Mortality of Critical Limb Ischemia 1 Year after Endovascular Revascularization in Patients with Type 2 Diabetes Mellitus

**DOI:** 10.1900/RDS.2022.18.20

**Published:** 2022-03-31

**Authors:** Em Yunir, Beta Agustia Wisman, Dono Antono, Arif Mansjoer, Angela Sarumpaet, Eni Iswati, Martha Rosana, Dicky L. Tahapary, Tri Juli Edi Tarigan, Pradana Soewondo

**Affiliations:** 1 Division of Endocrinology, Metabolism and Diabetes, Department of Internal Medicine, Dr. Cipto Mangunkusumo National General Hospital, Faculty of Medicine, Universitas Indonesia, Jakarta, Indonesia,; 2 Department of Internal Medicine, Dr. Cipto Mangunkusumo National General Hospital, Faculty of Medicine, Universitas Indonesia, Jakarta, Indonesia,; 3 Division of Cardiology, Department of Internal Medicine, Dr. Cipto Mangunkusumo National General Hospital, Faculty of Medicine, Universitas Indonesia, Jakarta, Indonesia.

**Keywords:** chronic limb ischemia, diabetes mellitus, endovascular revascularization, risk factors, survival

## Abstract

**BACKGROUND:**

Chronic limb ischemia (CLI) is strongly associated with increased mortality in diabetes patients.

**OBJECTIVE:**

The aim of this study was to evaluate factors affecting mortality within 1 year after endovascular revascularization in CLI patients.

**METHODS:**

This retrospective cohort study was based on medical records from the Integrated Cardiovascular Centre of Dr. Cipto Mangunkusumo National General Hospital, a tertiary care hospital in Jakarta, Indonesia. The study included 199 CLI patients with type 2 diabetes mellitus (T2DM) who underwent endovascular revascularization from January 2008 to June 2018. The patients were followed up for 1 year after endovascular revascularization. Kaplan-Meier and Cox proportional hazard analysis was used to analyze the data.

**RESULTS:**

1-year survival probability was 58.8%. Cox proportional hazard analysis showed that duration of diabetes (HR 3.52; 95% CI 1.34-9.22), anemia (HR 2.59; 95% CI 1.47-4.56), and smoking (HR 2.49; 95% CI 1.46-4.27) were significantly associated with mortality within 1 year after endovascular revascularization.

**CONCLUSIONS:**

In T2DM patients with CLI, duration of diabetes, anemia and smoking were associated with a higher risk of mortality within 1 year post endovascular revascularization

## 1 Introduction

Diabetic foot is a devastating diabetes complication, in which peripheral arterial disease (PAD) plays an important role [1]. Peripheral arterial disease affects approximately 202 million people globally, 28.7% of whom live in low-to middle-income countries [2]. The annual incidence of CLI ranges between 220 and 3000 new cases per million population [2]. 5-10% of patients with asymptomatic PAD develop CLI in 5 years [3]. Its widely known risk factors are smoking, hypertension, hypercholesterolemia, and diabetes [2]. Critical limb ischemia (CLI) is a severe manifestation of PAD; its clinical syndromes include ischemic pain at rest, non-healing ulcers, and gangrene [4].

CLI is associated with a high risk of amputation, cardiovascular events, and mortality [5]. Patients with CLI have mortality rates of 20% 5 years after diagnosis [6]. Endovascular revascularization is one of the treatment options for preserving limb function in patients with CLI [7].

A previous study showed that even after successful endovascular revascularization, the 5-year survival rate was only 23% [8]. A meta-analysis showed that endovascular revascularization is more feasible, safer, and more effective than open surgery [9]. Moreover, Simons et al. reported that advanced age, kidney failure, and functional impairment are associated with the highest hazard ratio in CLI after revascularization [10]. Several studies showed that the leading causes of death one year after revascularization were acute myocardial infarction (49.7%) and sudden death (10.1%) [11].

Multiple factors, including advanced age, chronic kidney disease (CKD), chronic heart failure (CHF), coronary artery disease (CAD), diabetes, and smoking, were found to be associated with higher mortality in patients with CLI following successful revascularization [10,12].

Soewondo et al. analyzed data from 18 diabetes centers in Indonesia and found that the average duration of diabetes was more than five years and 57.8% of patients experienced complications, including 16% with macrovascular complications [13]. Dyslipidemia was reported in 60% of patients with poor glycemic control (67-82%). A study conducted in Dr. Cipto Mangunkusumo National General Hospital as the national referral hospital in Indonesia showed that T2DM patients had comorbid coronary heart disease (46%), diabetes duration >5 years (73%), poor glycemic control with HbA1c >7% (71%), hypertension (61%), and uncontrolled LDL (85%) [14]. Moreover, patients with diabetic foot had the characteristics of anemia (90%) with comorbid hypertension (56.2%) and a mean duration of diabetes of 7 years [15]. These conditions may have influenced the outcome of endovascular revascularization in diabetic foot patients with severe PAD.

Furthermore, the procedure of endovascular revascularization for diabetic foot is rarely performed in tertiary hospitals in Indonesia. Therefore, this study is essential to understand the mortality and influencing factors in diabetic foot patients with CLI who undergo endovascular revascularization in Indonesia. Information obtained from this study is expected to be used as a reference for appropriate disease management of T2DM patients with CLI, improved performance of endovascular revascularization procedures, and optimized cost-effectiveness of hospitalization.

## 2 Methods

### 2.1 Subject and study design

This retrospective cohort study analyzed data obtained from the medical records of the Integrated Cardiovascular Centre Department of Dr. Cipto Mangukusumo National General Hospital in Jakarta, Indonesia. The study enrolled 288 T2DM patients diagnosed with CLI who underwent endovascular revascularization between January 2008 and June 2018. The inclusion criteria were:

- T2DM patients with foot complications
- Aged >18 years old
- PAD with CLI
- Underwent endovascular revascularization

Two hundred and eighty-eight patients were identified on the basis of the inclusion criteria. A total of 85 patients were excluded because they had incomplete medical record data, and 4 patients could not be contacted to be followed. The remaining 199 patients were included in the study. All patients were followed up 1 year after revascularization until the end of the study in June 2019 or death whichever came first. Telephone calls or home visits were made to clarify patients’ data. Endovascular revascularization was defined as any revascularization procedure using balloon angioplasty, drug-eluting balloon (DEB), and drug-eluting stent (DES). Ethical approval was obtained from the Health Research Ethics Committee of the Faculty of Medicineof the University of Indonesia as stated in letter no. 361/UN2.F1/ETIK/PPM.00.02/2019.

### 2.2 Measurements

Smoking was defined as active smoking, and any history of smoking. Cardiac comorbidities considered were CAD, heart failure, and arrhythmias. Anemia was defined as hemoglobin level <10 g/dl [16]. The study stated that preoperative hemoglobin <10 was associated with a significantly higher mortality <30 days after endovascular revascularization in women and men [16]. Hypertension was defined as blood pressure >140/90 mmHg or taking any hypertensive medications [17]. Dyslipidemia was defined as triglyceride level >150 mg/dl, or LDL >100 mg/dl, or HDL <40 mg/dl in men and <50 mg/dl in women [18] or taking anti-lipid drugs.

Chronic kidney disease was defined as estimated glomerular filtration rate (eGFR)<60 ml/min/1.73m^2^ within the last 3 months or more [19]. Computed tomography angiography (CTA) was performed to evaluate the location of stenosis. Duration of diabetes was defined with a separation at 10 years. Research conducted by Al-Rubeaan et al. states that patients with T2DM with a diabetes duration of more than ten years have an increased risk of death and more severe diabetic foot complications [20]. Based on the receiver operating characteristic (ROC) curve, neutrophil lymphocyte ratio (NLR)> 5.10 was taken as the cutoff point. To analyze 1-year mortality and stratify the risk of endovascular revascularization outcomes, we included any aortoiliac, femoropopliteal, infra- popliteal, and multiple stenosis which affected a long segment or more than two sites.

### 2.3 Statistical analysis

Statistical analyses were performed using IBM SPSS version 20 (SPSS, version 20.0; IBM Corp). Datawith a normal distribution were presented as mean value and standard deviation, whereas abnormal distribution data were presented as median and interquartile range. Kaplan Meier analysis was used to evaluate survival probability 1 year after endovascular revascularization. Cox proportional hazard was calculated to investigate the risk factors for CLI 1 year after endovascular revascularization.

## 3 Results

**Figure 1.**
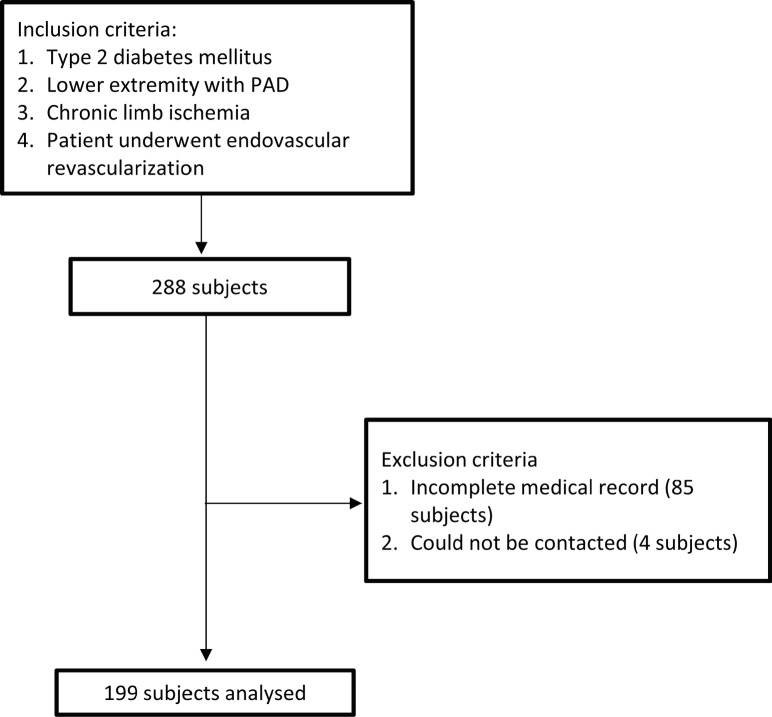
Subject recruitment process

Based on the recruitment process flow chart, there were 199 out of 288 T2DM-CLI patients who underwent endovascular revascularization and who met the inclusion criteria (**Figure 1**). **Table 1** shows the baseline characteristics of the study participants. The average age of the participants was 62.9 ± 10.1, and 47.2% were men. Notable baseline characteristics were median duration of diabetes at 9 (6-15) years and approximately half of the subjects smoked (45.2%) and had hypertension (70.4%). The most frequent stenosis sites were femoropopliteal (59.3%) and infra-popliteal (26.6%). Balloon angioplasty was the most common procedure (72.4%). The proportion of mortality within 1 year after revascularization was 41.2%.

**Table 1.** Demographic and clinical characteristics

Variable	Value (n =199)
Men, n (%)	94 (47.2)
Age (years), mean (SD)	62.9 (10.1)
Duration of diabetes (years), median (IQR)	9 (6-15)
Cardiac comorbidity, n (%)	94 (47.2)
Smoking, n (%)	90 (45.2)
Hypertension, n (%)	140 (70.4)
Neuropathy, n (%)	149 (74.9)
Arterial stenosis location	
Aortoiliac, n (%)	9 (4.5)
Femoropopliteal, n (%)	118 (59.3)
Infrapopliteal, n (%)	53 (26.6)
Multiple, n (%)	19 (9.5)
Albumin (mg/dl), median (IQR)	3.1 (2.5-3.9)
Hemoglobin (mg/dl), mean (SD)	10.6 (2.0)
NLR, median (IQR)	5.0 (2.9-9.1)
HbA1c (%), median (IQR)	7.3 (6.8-9.0)
eGFR (ml/min/1.73m^2^), median (IQR)	62.5 (38.0-91.0)
Length of stay (days), median (IQR)	15 (3-28)
Type of endovascular revascularization	
Balloon angioplasty, n (%)	144 (72.4)
Drug-eluting balloon (DEB), n (%)	37 (18.6)
Drug-eluting stent (DES), n (%)	18 (9.0)
1-year mortality, n (%)	82 (41.2)

**Legend**: NLR: neutrophil lymphocyte ratio; eGFR: estimated glomerular filtration rate; HbA1c: hemoglobin A1c

**Figure 2.**
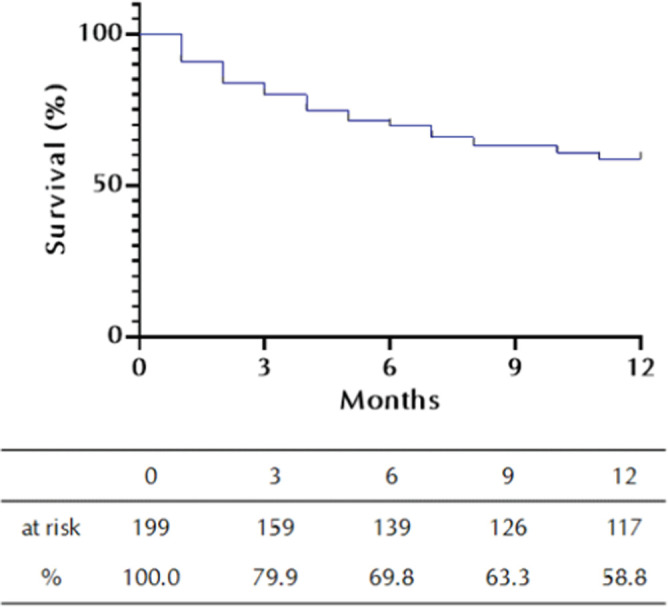
Kaplan-Meier life table analysis of mortality in critical limb ischemia (CLI) patients within 1 year after endovascular revascularization

Kaplan-Meier analysis showed that the patient survival probability within 1 year after endovascular revascularization was 58.8%. Most deaths occurred in the first 3 months after the procedure (**Figure 2**).

**Figure 3.**
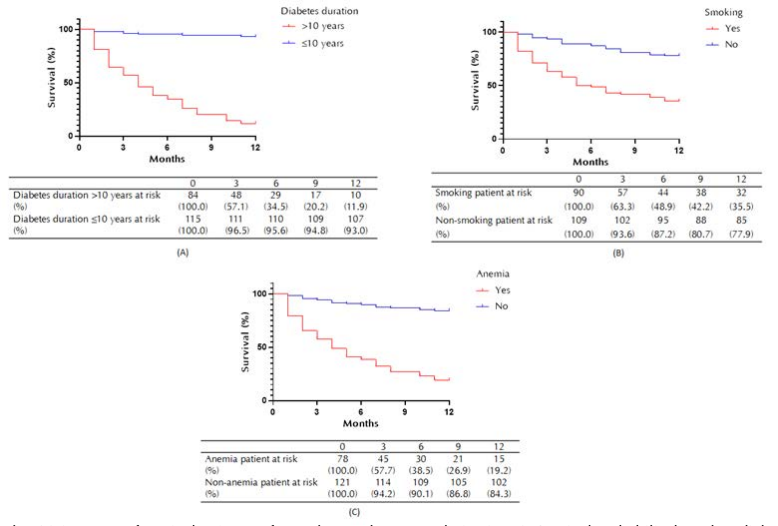
Kaplan-Meier curve of survival at 1 year after endovascular revascularization. A: Survival probability based on diabetes duration (11.9% vs. 93.0%). B: Survival probability based on smoking status (35.5% vs. 77.9%). C: Survival probability based on anemia status (19.2% vs. 84.3%).

Bivariate analysis showed that age, duration of diabetes, smoking, cardiac comorbidity, hypertension, neuropathy, dyslipidemia, eGFR, HbA1C, albumin level, and neutrophil lymphocyte ratio were associated with mortality within one year after endovascular revascularization (**Table 2**). Meanwhile, multivariate analysis showed that duration of diabetes (HR 3.517; 95% CI 1.341-9.22), smoking (HR 2.49; 95% CI 1.464.27), and anemia (HR 2.59; 95% CI 1.47-4.56) increased the mortality risk within 1 year after endovascular revascularization (Table 2). Kaplan-Meier analysis revealed the survival probabilities for duration of diabetes, smoking status, and anemia within 1 year after endovascular revascularization. The majority of patients with diabetes duration >10 years, smoking, or anemia died within 3 months after endovascular revascularization (**Figure 3**).

**Table 2.** Predictors of mortality after endovascular revascularization

Variables	Bivariate analysis	Multivariate analysis		
	p-value	HR	95% CI	p-value
Sex	0.326	-	-	-
Age	0.004^*^	0.89	0.49-1.60	0.69
Duration of diabetes > 10 years	<0.001^*^	3.52	1.34-9.22	0.011^*^
Smoking	<0.001^*^	2.49	1.46-4.27	0.001^*^
Hypertension	<0.001^*^	5.39	0.66-44.09	0.12
Cardiac comorbidity	<0.001^*^	1.16	0.64-2.09	0.62
Neuropathy	<0.001^*^	1.77	0.79-3.98	0.16
Dyslipidemia	<0.001^*^	3.93	0.48-31.95	0.20
Chronic kidney disease	<0.001^*^	1.69	0.82-3.48	0.16
Anemia	<0.001^*^	2.59	1.47-4.56	0.001^*^
HbA1c > 7%	<0.001^*^	1.77	0.71-4.39	0.22
Albumin level < 2.5 g/dl	<0.001^*^	1.65	0.99-2.73	0.053
NLR > 5.10	<0.001^*^	1.73	0.91-3.31	0.09
Stenosis location				
Aortoiliac	Ref	-	-	-
Femoropopliteal	0.813	-	-	-
Infrapopliteal	0.969	-	-	-
Multiple	0.870	-	-	-

**Legend:** All variables from bivariate analysis where p <0.25 were included in the multivariate analysis. Cox proportional hazard analysis was used in bivariate and multivariate analysis.

* Significance p <0.05. Abbreviations: eGFR: estimated glomerular filtration rate; NLR: neutrophil lymphocyte ratio; HR: hazard ratio; CI: confidence interval.

## 4 Discussion

This study aimed to investigate the factors affecting the mortality of T2DM patients with CLI one year after endovascular revascularization. Our study showed that the survival probability for T2DM patients with CLI one year after endovascular revascularization was 58.8%. Duration of diabetes, anemia, and smoking were the significant predictors of mortality.

The study revealed that duration of diabetes significantly increased the risk of mortality in T2DM patients with CLI after endovascular revascularization. Diabetes-associated atherosclerosis can lead to complications in all major vascular beds and become prevalent prior to the diagnosis of diabetes; its severity increases with worsening blood glucose control and increasing duration of diabetes. Individuals with longer diabetes duration are usually associated with greater risk of diabetic foot ulcer (DFU) because of the hyperglycemic condition [21-23]. Several studies showed that T2DM is a predictor for poor outcome of cardiovascular events. A meta-analysis conducted by Vrsalovic et al. stated that diabetes increases the risk of mortality in patients with PAD (OR 1.89; 95% CI 1.51-2.35, p<0.001) [24]. Duration of diabetes was associated with the risk of micro- and macrovascular complications [25].

CLI involves macro- and microvascular defects that lead to reduced arterial perfusion, causing decreased oxygen requirement and nutrient supply in the downstream tissue. Cortez et al. similarly reported that the risk of complications such as amputation, diabetic foot, nephropathy, diabetic retinopathy, acute myocardial infarction, and cerebrovascular accidents was associated with duration of diabetes>10 years (32%) compared to <5 years (12.1%) [26]. Furthermore, previous reports detected several complications that lead to increased limb loss and mortality in patients with CLI [27]. However, Oberto et al. suggested that both patients with and those without diabetes had similar outcomes of clinical improvement after stent revascularization techniques [28]. Despite evidence of improvement in endovascular revascularization, patients with cardiovascular risk and diabetes contribute to accelerated atherosclerosis and high risk of premature death [29].

Moreover, similar to other reports of risk factors for mortality in patients at cardiovascular risk, anemia was a significant predictor of death within 1 year of endovascular revascularization (HR 2.59; 95% CI 1.474.56). Anemia contributes to severe complications and increased mortality as observed in the clinical setting. A significant level of in-hospital death in anemic patients has been observed in large cohort studies of both cardiac and non-cardiac surgery [30]. Moreover, anemia is more commonly seen in chronic inflammatory states that mediate increasing levels of plasma cytokines such as tumor necrosis factor, interleukin-1, and interferons, which may contribute to increased mortality [31].

The Quality Improvement Program (NSQIP) dataset showed that anemia increased the rates of perioperative infection and mortality in non-cardiac surgical patients [32]. A meta-analysis conducted by Yammine et al. detected a significant association between diabetic foot ulcer healing and hemoglobin level [33]. In a similar study based on the French COhorte des Patients ARTériopathes (COPART) registry, which included more than 900 patients with PAD undergoing surgery for CLI, anemia was found to be an independent factor for postoperative death and major amputation [16]. Costa et al. found that hemoglobin levels lower than 11 g/dl are an important independent factor for major amputation and death (OR 5.5 and 4.0, respectively, p<0.001) [34]. Low levels of hemoglobin in DFU reduce oxygen transport capacity and affect erythrocyte deformability causing impaired microcirculation. This is a vicious cycle leading to prolonged wound recovery [35].

This study found that smoking was significantly associated with mortality 1 year after endovascular revascularization in T2DM patients. Smoking is one of the risk factors for developing atherosclerosis and increases the risk of progression towards CLI [36]. People who smoke 1-10 cigarettes per day have a significantly higher rate of disease progression and major adverse cardiovascular events (MACEs) [37]. The Bypass versus Angioplasty in Severe Ischemia of the Leg (BASIL) trial showed worse survival rates for both current and ex-smokers compared to non-smokers [6]. This result was supported by a meta-analysis of prospective studies showing that smoking increases graft failure 1.99 fold (95% CI 1.6-2.47, p<0.001) [38]. Indeed, continued smoking after revascularization causes approximately a threefold increased risk of graft failure. Even though we did not specify the characteristics of daily and occasional smoking among the participants, the mortality risks of non-daily smokers and daily smokers were similar [37]. Cigarette smoking is also an independent risk factor for diabetic neuropathy caused by the mechanism of oxidative stress due to alterations of nitric oxide concentrations, which lead to cellular damage [39].

There is a scarcity of studies investigating the efficacy of endovascular revascularization in T2DM patients with CLI. Therefore, research on risk factors affecting decision-making in relation to revascularization procedures should be extended. As this study was retrospective, we were limited to variables that were collected during the treatment period and unable to assess additional factors which have not been recorded. Moreover, several data were still available as paper-based records, which limited data collection additionally.

## 5 Conclusions

This study demonstrated that T2DM patients with CLI face a high risk of mortality and poor prognosis one year after endovascular revascularization. Moreover, duration of diabetes, anemia, and smoking are associated with poor survival after revascularization. Improving life expectancy should be targeted as well as, if not more than, limb saving. Future studies should apply foot risk screening and stratification methods and include an analysis of the correlation with severity of disease and response to treatment to determine survival probability and factors affecting mortality, particularly in different time frames.

## Abbreviations

BASIL: Bypass versus angioplasty in severe ischemia of the leg

CAD: coronary artery disease

CHF: chronic heart failure

CKD: chronic kidney disease

CLI: chronic limb ischemia

COPART: French COhorte des Patients ART ériopathes

CTA: computed tomography angiography

DEB: drug-eluting balloon

DES: drug-eluting stent

eGFR: estimated glomerular filtration rate

MACEs: major adverse cardiovascular events

PAD: peripheral arterial disease

ROC: receiver operating characteristic

T2DM: type 2 diabetes mellitus
